# A cross-sectional study addressing the importance of work and other everyday activities for well-being among people with mental illness: does additional vulnerability matter?

**DOI:** 10.1186/s12888-021-03388-8

**Published:** 2021-07-31

**Authors:** Lisa Eklund, A. Birgitta Gunnarsson, Jan-Åke Jansson, Parvin Pooremamali, Mona Eklund

**Affiliations:** 1 Department of Sociology, Lund University, Lund, Sweden; 2 Department of Research and Development, Region Kronoberg, Växjö, Sweden; 3 Department of Clinical Neuroscience and Rehabilitation, University of Gothenburg, Gothenburg, Sweden; 4 Department of Psychology and Department of Health Sciences, Lund University, Lund, Sweden; 5 Department of Community Medicine and Rehabilitation, Occupational Therapy, Umeå University, Umeå, Sweden; 6 Department of Health Sciences, Lund University, P. O. Box 157, SE-22100 Lund, Sweden

**Keywords:** Activity, Mental illness, Participation, Recovery, Vulnerability, Well-being, Work, Quality of life

## Abstract

**Background:**

Work and other everyday activities are beneficial for well-being among people with mental illness, but poor circumstances can create detrimental effects, possibly aggravated by additional vulnerabilities linked with their mental illness. This study aimed to investigate how activity factors were related to well-being and functioning among three vulnerable groups using outpatient mental health care – young people with psychosis, people with a history of substance use disorder (SUD), and immigrants with post-traumatic stress disorder (PTSD) – while controlling for vulnerability group, age and gender.

**Methods:**

Participants represented the three types of vulnerability (*n* = 46/57/39). Data collection, using self-report and interviewer-rated questionnaires, concerned aspects of everyday activity (work experiences; views of the worker role; satisfaction with everyday occupations; activity level), well-being (quality of life: life and health; quality of life: environmental aspects; recovery) and functioning (psychosocial functioning; symptom severity). Spearman correlations and General Linear Modelling were used.

**Results:**

Activity satisfaction was positive (*p* < 0.001) but recent work experience negative (*p* = 0.015) for the life and health aspect of quality of life. Activity satisfaction was positive for the environmental aspects of quality of life (p < 0.001). Resources for having a worker role (p < 0.001) and belief in having a future worker role (*p* = 0.007) were positively associated with better recovery. Activity level (*p* = 0.001) and resources for having a worker role (*p* = 0.004) showed positive associations with psychosocial functioning. Belief in a future worker role (*p* = 0.011) was related with symptom level. Women had less severe symptoms in the young group with psychosis. Regarding vulnerability group, young people with psychosis perceived better quality of life; those with a history of SUD had less severe psychiatric symptoms; and the recent immigrants with PTSD had the highest level of psychosocial functioning.

**Conclusion:**

Work experience may not be conducive to well-being in itself; it is satisfaction with work and other activities that matters, and worker and employer expectations need alignment. No vulnerability group seemed consistently more disadvantaged regarding well-being and functioning, but the fact that differences existed is vital to acknowledge in activity-based rehabilitation. Inquiring about meaningful activities and providing opportunities for executing them would be a fruitful way of support.

**Supplementary Information:**

The online version contains supplementary material available at 10.1186/s12888-021-03388-8.

## Introduction

Researchers theorize that engaging in everyday activities is an essential human need [1]. Work, which is one aspect of everyday activity, has been established for a long time as being generally important for human well-being and it has been proposed that working is at the core of human life [2]. Unemployment is associated with poorer mental health outcomes [3, 4], which makes access to work important for people with mental ill-health. However, not all aspects of work or every type of work enhance well-being. Working conditions are decisive for well-being and productivity, which may deteriorate quickly under poor working conditions [5]. Research has also shown that the way people with mental illness perceive their work situations is related to subjective health and well-being factors. The many positive connotations, such as contributing to society, providing for one’s family, and enjoying being in a community of co-workers [6, 7], dissipate if work is seen as demanding, stressful, and without a certain degree of flexibility that may be necessary to handle fluctuations in one’s state of mental health [8, 9].

For those who do not have employment – involuntarily or not – other forms of everyday activities may partly serve as a substitute and provide a sense of being engaged in productive activities, given that the activities are seen as meaningful [7]. Taking care of one’s home, developing social activities, and participating in voluntary work are examples of activities that may be important and meaningful per se, but also shape routines that support employment. Both salaried work and other everyday activities can bring experiences such as belonging in a social context, contributing to society, developing skills that may be of use to others, doing something others can appreciate, and feeling proud of an accomplishment [6, 7, 10]. Knowing more about how work, and everyday activities in general, are related with perceived well-being may be useful when developing work-oriented support for people with mental illness, an area identified as a critical challenge by the Organisation for Economic Co-operation and Development (OECD) [4].

The labor market is a field, however, where stigmatizing attitudes may create detrimental effects [11]. Such attitudes may entail worse consequences than the illness in itself [12] and are typically based on ignorance and are expressed in terms of prejudiced and negative attitudes, and excluding or avoiding behaviors [13]. People with mental illness do not, however, constitute a homogenous group, and stigma may concern not only mental illness, but also various other attributes that deviate from dominant norms in society such as poverty, race, low education, addiction, and ethnic origin [14–16]. Living with mental illness does thus not mean the same to everyone, and other characteristics are likely to shape different types of vulnerabilities. The fact that vulnerability is shaped by both internal and contextual factors was acknowledged by Mackenzie and colleagues [17] and on a similar note Alvarez-Castillo and colleagues defined vulnerability as “complex and fluid. It is complex because it is the result of interacting factors and situations /.../ Vulnerability is fluid because it is not the inevitable consequence of biological circumstances but of power relations that are embedded in relationships, norms, practices, and policies” [18 , p. 130]. Vulnerability is thus a complex interplay among a variety of inherent, situational and pathogenic sources, and with respect to people with mental ill-health the causes may lie in personal, social, economic, political and environmental factors, including a social structure characterized by injustice, discrimination, oppression, abuse and stigma.

The focus of this study is to explore whether differences in type of vulnerability among people with mental illness play a role in relationships between factors related to work, other everyday activities and aspects of well-being and functioning. Such relationships have not been investigated in previous research, and adding more knowledge in this regard was the incentive for the current study. Vulnerability is here specified as the additional difficulties that subgroups of people with mental illness may experience as a consequence of having a certain type of mental illness. This study, undertaken in Sweden, puts three groups with mental illness in focus. One group consists of people who develop psychosis at a young age and who as a consequence have fewer career opportunities than others of the same age. Their vulnerability also oftentimes includes being unable to proceed to higher education, and entrance to the labor market is made difficult [19, 20]. They typically report low self-esteem, stigmatizing attitudes from others, and difficulties in identifying and achieving goals as hindering factors in relation to work and employment [21]. A second group is formed by people with a history of substance use disorder (SUD) in addition to mental illness. A combination of SUD and mental illness entails vulnerability in terms of difficulties in gaining and keeping employment, and the situation of having or not having employment seems to reinforce the current situation, creating a vicious circle [22]. Moreover, concurrent substance use influences duration of mental illness and treatment outcomes negatively [23]. This group is also highly expose to stigma [24]. A third group with particular vulnerability are immigrants with mental illness who have recently fled their country of origin and try to settle in a new cultural context [25]. Many immigrants in Sweden originate from the Middle East, and research addressing those who come from that region and have a mental illness has shown that they experience great difficulties in finding a job or other meaningful activities and that they are dissatisfied with the available support [26]. Many of these receive a diagnosis of post-traumatic stress disorder (PTSD). Although these three groups have in common that they often receive attention in media and policy debates in Sweden, there are also substantial differences between them regarding age, cultural backgrounds and possible stigmatizing attitudes from the environment. These diversities make them interesting subgroups for the present study.

The study centers on everyday activities, including aspects of work and other productive activities, and how these are associated with well-being and functioning in the three selected subgroups of people with mental illness. Quality of life and personal recovery have been highlighted as important outcomes in mental health care services in recent years, in addition to medically oriented outcomes such as functioning and symptom severity. This was the rationale for selecting these variables as correlates in relation to the activity factors. Although it is well established that there is a relationship between engagement in everyday activities, including work, and factors pertaining to well-being and functioning [1, 6, 7], much remains to be known. Whether such relationships are similar or vary among the three target groups in this study will contribute with valuable knowledge for how to devise tailored support in mental health and social services. Moreover, researchers have proposed that there may be gender-specific pathways to quality of life and well-being for women and men [27, 28]. Whether work, employment and other activity factors have gender-specific impacts on well-being and functioning is another research question that warrants attention, given that research is showing that gender-based vulnerabilities exist as a consequence of differences in opportunities, status and rights between men and women [18]. There are also gendered differences in vulnerability in relation to mental illness. Women generally report higher incidences of psychiatric and stress-related disorders [29] while men have earlier onset and higher incidences of schizophrenia and SUD [30]. According to the World Health Organization [31], this may at least partly be due to socially constructed differences in roles, status and power that interact with biological differences between women and men, thus contributing to differences in the nature of mental health problems, health seeking behaviors, and responses of the mental health services. At the same time, having paid work may be more crucial to well-being and health among men due to male breadwinner ideals, which would imply that there is an association between work and sense of self-worth that is stronger among men (Artazcoz et al. 2004). However, the influence of gender on the relationship is not absolute and has been shown to depend on gender regime, that is, whether women and men are similarly attached to the labor market or not. Unemployment in a gender regime characterized by the ambition of gender equity, such as in Sweden, seems to affect women’s and men’s mental health equally [32], although this may not necessarily be so for individuals who have recently settled in Sweden and originate from gender regimes where men are main breadwinners.

The aim of this study was to investigate how activity factors, both salaried work and other everyday activities, were related to well-being and functioning among three vulnerable groups with mental illness – young people with psychosis, people with a history of SUD, and newly arrived immigrants with PTSD – while controlling for vulnerability group, age and gender.

## Methods

This cross-sectional study, performed in Sweden, was based on convenience sampling as further described below. It followed all pertinent ethical guidelines and was approved by the Regional Ethical Review Board in Lund, Sweden, Reg. Nos. 2015/357 and 2017/137.

### Sampling procedure

Settings in three different care and support contexts were approached to identify eligible participants. All were located in the southern counties of Sweden. Psychosis units in two counties were contacted to identify young people with psychosis. Six units, out of seven approached, agreed to collaborate with the research team. People with SUD were identified through contacts with the authorities in a larger town, where the social services ran a support service for this target group. Those who had recently immigrated were sought through a center for people with PTSD that offers education in Swedish, other lessons, and opportunities for participating in vocational training in various workshops.

The procedure for selecting participants was the same in all three contexts. A gatekeeper among the staff served as a contact person for the research team and identified eligible clients. Inclusion criteria were a mental illness and the additional characteristic distinguishing the respective groups. An additional eligibility criterion was sufficient language skills in Swedish or English to take part in the data collection. In the case of newly arrived immigrants with PTSD, alternative possible languages were Arabic, Farsi, Pashtu, and Dari. The gatekeeper contacted those who fulfilled these criteria and provided written and oral information about the project. Those who accepted to participate gave their written informed consent and provided their contact details. The gatekeeper organized the appointments for data collection, which was performed by one of the researchers or research assistants in a secluded room in the setting where the participant received their mental health support.

### The participants

Based on a power analysis and using the SDO instrument (see below), we strove to include 60 participants from each group. With that number we would be able to detect a group difference on the SDO of 0.5 points with 80% power at *p* < 0.05. As seen in Table 1, which presents some characteristics of the participants, we were not able to achieve that number. Reasons for this included reorganization in the PTSD center and difficulties in identifying young people with psychosis for whom a diagnosis had been made, as it is common to await development before making such a severe diagnosis as psychosis. Table 1 also indicates some differences between the groups, which for natural reasons included age, diagnosis, being foreign-born or not, and having children or not. There was also a clear difference regarding recent engagement in employment or regular studies. The many language options did not restrict participation to a specific region of origin for the immigrants with PTSD, but it turned out that all of them came from countries in the Middle East (Afghanistan, Syria, Iraq, Lebanon, Yemen, and Palestine). The information regarding education confirms that the group of young people with psychosis were disadvantaged in relation to higher education, compared to the other groups. Significantly fewer in the SUD group were married or cohabiting, despite this group being older than the young people with psychosis. The group of immigrants with PTSD more frequently reported physical problems than the other groups, mainly related to pain, such as migraine and back and shoulder pain. The other groups reported more diverse physical complaints, for example, overweight, leg pain, and eczema in the young group with psychosis and arthritis, asthma, and heart problems in the SUD group.

**Table 1:** Sociodemographic characteristics of the participants

	Young people with psychosis *N* = 46	History of SUD *N* = 57	Immigrants with PTSD *N* = 39	*P*-value
Age; mean (SD) years	26 (3)	43 (9.7)	41 (9.5)	< 0.001
Sex; % women/men/non-binary	30/67/2	29/71	33/67	ns.
Foreign-born; %	22	10	100	< 0.001
Type of household; %				< 0.001
Single	52	89	18	
Married or living with partner	17	9	80	
Living with family or friends	30	2	3	
Having children; %	15	49	80	< 0.001
Highest education; %				< 0.001
Completed 9-year school or lower	41	51	77	
Completed high school	59	40	8	
Completed university degree	–	9	15	
Employment or regular studied for at least 2 months the past 2 years; %	46	5	97	< 0.001
Self-reported diagnosis; %				< 0.001
Psychosis	89	4	0	
Neuropsychiatric disorder	2	35	0	
Mood/anxiety disorder	2	14	16	
PTSD	0	5	83	
Other	7	42^1)^	0	
Experiencing physical problems; %	30	48	81	< 0.001

^1)^ Included here are 40% in the group with a history of SUD who did not report a diagnosis or reported that they did not have one

### Data collection

Those who performed the data collection had long experience from working with people with mental illness and were educated within occupational therapy or psychology. A background questionnaire was developed for the study and included questions regarding socio-demographics, medication, self-reports regarding diagnosis and whether or not the participant had physical problems. These variables were only used to describe the samples and were not included in further analyses. Additional instruments, as specified below, were used to address variables pertaining to everyday activities, well-being, and level of functioning.

#### Everyday activities

Two instruments were used to address employment, work, and other everyday activities. One was the Worker Role Self-assessment (WRS) [33, 34], which is a self-administered questionnaire with statements addressing one’s beliefs in having a future worker role and one’s resources for having a worker role. A 16-item version was used. The response scale has five alternatives, from 1 = “totally disagree” to 5 = “totally agree”. A higher rating indicates stronger beliefs/resources. WRS has shown to form two subscales (“beliefs in a future worker role” and “worker role resources”) with good construct and known-groups validity and satisfactory homogeneity [34]. The items that formed beliefs in a future worker role concerned, e.g., believing in a future working life for oneself, having goals for what to accomplish in working life, seeing the worker role as important, and getting support from family and services to gain a worker role. Items pertaining to resources for having a worker role concerned, e.g., awareness of skills and limitations, taking responsibilities, having good routines, and being flexible.

Satisfaction with Daily Occupations (SDO) is a screening tool that targets four domains of everyday activities – work/studies, leisure, home management, and self-care [35–37]. A version with 14 items was used, and each item includes two questions. The first asks whether one currently performs the exemplified activity or not and has a yes/no response scale. The second question asks about one’s satisfaction with the exemplified activity, regardless of whether the response to the first question is yes or no. It is thus possible to be satisfied with not currently being involved in an activity, and vice versa. The satisfaction rating has a seven-point response scale, from 1 = “worst possible satisfaction” to 7 = “best possible satisfaction”. The yes/no responses may be summed into an activity level score (possible range 0–14) and the satisfaction score into a satisfaction with everyday occupations score (possible range 14–98). The SDO has shown good construct validity, homogeneity and test-retest stability [35–37].

Two SDO items from the work/studies domain (being employed or admitted to an education at any point during the past 2 months, and currently involved in work or studies) were further used to indicate recent work/study experience (possible range 0–2) and satisfaction with that experience (possible range 2–14). These two items thus form a subset of the SDO items, and the reason for including both total SDO scores and sub-scores for the work items was to test if work specifically, or of everyday activity as a whole, was the most important factor for the selected well-being and functioning variables.

#### Well-being

Two aspects of well-being were addressed; quality of life and personal recovery.

The quality of life instrument used was the Manchester Short Assessment of Quality of Life (MANSA) [38]. A Swedish version, used for the current study, has shown good construct validity and homogeneity [39]. MANSA includes 12 items tapping subjective aspects of life satisfaction. A seven-point repose scale is used, ranging from 1 = “could not be worse” to 7 = “could not be better”. Higher scores thus indicate better quality of life. A recent study concluded that the items form two factors [40]. One concerns “life and health-related aspects”, encompassing items pertaining to life as a whole, mental health, physical, leisure activities, friendships, employment situation, financial situation, and sex life. The second factor, termed “quality of environment”, was formed by items addressing living with someone (or not), family relationships, accommodation, and personal safety. Acceptable homogeneity (omega = 0.78/0.68) was found for these factors. The Swedish version has also been found to form two factors [41]. Although the homogeneity estimate was a bit low for the second factor, we decided to treat MANSA as consisting of two factors in the analyses in this study.

The second aspect of well-being addressed was personal recovery, defined as a subjective journey towards personal growth and finding new meaning and hope in life despite remaining psychiatric symptoms [42]. The measure used was the Swedish version of Questionnaire about the Process of Recovery (QPR), which consists of 16 items, formulated as statements [43]. The Swedish version. Examples of item contents are “my life has a purpose”, “can develop relation to others”, “can take control of my life”, and “can see the positive things I’ve done”. The response scale has five anchors from 1 = “largely disagree” to 5 = “largely agree” and a higher score denotes a higher level of personal recovery. The Swedish version has shown good internal consistency reliability and adequate construct validity [43].

#### Level of functioning

The Global Assessment of Functioning (GAF) instrument [44] is performed by a clinically skilled professional and may be used to generate two ratings; symptom level and psychosocial functioning. Both symptom level and psychosocial functioning are rated on an interval from 0 to 100, where a higher score indicates less severe symptoms and better psychosocial functioning, respectively. GAF scores have been found reliable with only brief training of the professionals [45]. Those who performed the ratings in the current study received training based on realistic video cases and subsequent calibration against a skilled and experienced GAF rater. The instrument is widely used and has shown adequate reliability and validity [46].

### Data analyses

Non-parametric tests were used where possible since most variables were of ordinal type. Spearman correlations were used in order to analyze how activity factors were associated with aspects of well-being and functioning. The strength of associations was estimated in accordance with Cohen [47], who proposed that correlations < 0.30 are weak, 0.30–0.50 are moderate and > 0.50 are strong. Univariate General Linear Modelling (GLM) was then performed with the aim of taking the influence of age, vulnerability group and gender into account. Each of the well-being and functioning variables were set as the dependent variable in the GLM models. The activity variables were entered simultaneously as covariates to identify the strongest contributor(s) to the well-being factor at target. Correlations among the covariates (the activity variables) were calculated to check for collinearity. The correlations varied between r_s_ = 0.053 and r_s_ = 0.578 and four of them exceeded 0.5. Two of these concerned recent work experiences (correlated at r_s_ = 0.578 with satisfaction with work experience and at r_s_ = 0.542 with activity level), one concerned satisfaction with work experience (correlated at r_s_ = 0.524 with activity satisfaction), and one concerned belief in a future worker role (correlated at r_s_ = 0.568 with resources for having a worker role). The remaining 11 associations between covariates were very low or moderate in size, and we estimated that inter-correlations between covariates did not indicate a problem of collinearity. The group variables (gender and type of vulnerability group) were, first one at a time and then simultaneously, set as fixed factors to identify their possible influence on the relationship between everyday activities and well-being/functioning. Test of between-subjects effects were supplemented with parameter estimates with robust standard errors. A *p*-value of < 0.05 was set for statistical significance. The software used was the IBM SPSS statistics 26.0 [48].

## Results

### Associations of work and other activity factors to well-being and functioning

Descriptives for all result variables are shown in Table 2. Work/study experience, in terms of having had employment or regular studies for at least 2 months during the past 2 years, was positively associated with the research assistant’s rating of psychosocial functioning, and negatively associated with that of symptom level (see Table 3). Satisfaction with one’s work situation was positively related with the quality of one’s life and health, the quality of one’s environment, and psychosocial functioning. Belief in a future worker role was positively associated with all well-being variables (both quality of life aspects and personal recovery) and with symptom level, but not with psychosocial functioning. The association with personal recovery was particularly strong. Similarly, rating one’s resources for having a worker role high was associated with high ratings on all well-being and functioning variables except for the quality of one’s environment. The association with personal recovery was again strong. Activity satisfaction was positively associated with all well-being and functioning variables except for symptom level, and the relationship with quality of one’s life and health was strong. Activity level was positively related with the quality of one’s life and health, personal recovery, and psychosocial functioning.

**Table 2:** Descriptives for the result variables; mean (SD)

	Young people with psychosis *N* = 46	History of SUD *N* = 57	Immigrants with PTSD *N* = 39
Recent work/study experience	1 (0.9)	0.2 (0.5)	1.9 (0.2)
Satisfaction with work/study experience	9.3 (3.2)	6.7 (3.6)	10.7 (3.4)
Belief in a future worker role	30.4 (6)	28.5 (6.5)	28.5 (5.2)
Resources for a worker role	27.8 (5.6)	29.7 (5)	30.3 (4.7)
Activity satisfaction	70.5 (11.5)	60 (18.4)	63.7 (15.1)
Activity level	8.5 (2.2)	7.5 (2.2)	10.6 (2.5)
Quality of life; life and health	35.5 (8)	29.9 (10.6)	28.1 (7.9)
Quality of life; environment	21.9 (4.9)	18.5 (5.2)	20.3 (4.8)
Personal recovery	60.7 (11)	62.1 (9.5)	62.4 (8.1)
Psychosocial functioning	56.9 (9.4)	60.5 (5.6)	63.3 (9.2)
Symptom level	57.7 (9)	61.9 (6.2)	56.6 (7.9)

**Table 3:** Correlations between work and other everyday activity factors and variables pertaining to well-being and functioning, based on the sample as a whole (*N* = 142)

	Quality of life; life and health	Quality of life; environment	Personal recovery	Psychosocial functioning	Symptom level
Recent work/study experience	r_s_ = 0.046; ns.	r_s_ = 0.136; ns.	r_s_ = 0.145; ns.	r_s_ = 0.238; *p* = 0.005	r_s_ = −0.190; *p* = 0.025
Satisfaction with work/study experience	r_s_ = 0.325; p < 0.001	r_s_ = 0.337; p < 0.001	r_s_ = 0.158; ns.	r_s_ = 0.181; *p* = 0.034	r_s_ = −0.130; ns.
Belief in a future worker role	r_s_ = 0.434; p < 0.001	r_s_ = 0.270; *p* = 0.001	r_s_ = 0.606; p < 0.001	r_s_ = 0.130; ns.	r_s_ = 0.215; *p* = 0.011
Resources for a worker role	r_s_ = 0.256; *p* = 0.003	r_s_ = 0.072; ns.	r_s_ = 0.705; p < 0.001	r_s_ = 0.280; *p* = 0.001	r_s_ = 0.218; *p* = 0.010
Activity satisfaction	r_s_ = 0.575; p < 0.001	r_s_ = 0.456; p < 0.001	r_s_ = 0.264; *p* = 0.002	r_s_ = 0.189; *p* = 0.028	r_s_ = 0.116; ns.
Activity level	r_s_ = 0.292; *p* = 0.001	r_s_ = 0.152; ns.	r_s_ = 0.248; *p* = 0.004	r_s_ = 0.428; p < 0.001	r_s_ = −0.070; ns.

### Relationships according to GLM models

Findings from the GLM analyses are presented schematically in Table 4 and are described in more detail below. For each dependent variable, the GLM includes an initial model (step 1) with all activity factors entered simultaneously. The second step adds the factors we wanted to control for (age, vulnerability group and gender) one at a time to the initial model. The third step consists of the initial model plus age, vulnerability and gender entered together.

**Table 4:** Schematic summary of activity variables of relevance to well-being and functioning according to the GLM models

	Quality of life; life and health	Quality of life; environment	Personal recovery	Psychosocial functioning	Symptom level
Step 1. Initial model	Activity satisfaction; *Recent work experience*	Activity satisfaction	Resources for having a worker role; Belief in a future worker role	Activity level; Resources for having a worker role	Belief in a future worker role
Step 2. Controlling for age	Activity satisfaction; *Recent work experience*	Activity satisfaction	Resources for having a worker role; Belief in a future worker role	Activity level ^a^	Belief in a future worker role
Controlling for vulnerability group	Activity satisfaction; *Recent work experience;* Activity level ^b^	Activity satisfaction	Resources for having a worker role; Belief in a future worker role	Activity level; Resources for having a worker role ^b^	Resources for having a worker role ^b^
Controlling for sex	Activity satisfaction; *Recent work experience*	Activity satisfaction	Resources for having a worker role; Belief in a future worker role	Activity level; Resources for having a worker role	Belief in a future worker role
Step 3. Controlling simultaneously for age, vulnerability group, and sex	Activity satisfaction; *Recent work experience* ^b^	Activity satisfaction	Resources for having a worker role; Belief in a future worker role	Activity level	All activity variables were non-significant ^c^

*Note*: Italics indicate a negative association to the target variable

^a^Age significant in the model

^b^Vulnerability group significant in the model

^c^Interaction effect between group and sex

#### Quality of life: life and health

Rating one’s activity satisfaction high was positive (F = 24.05; B = 0.271; *p* < 0.001) for one’s quality of life and health in step 1, whereas recent work experience was a negative factor (F = 6.12; B = -2.548; *p* = 0.015).

Age did not become statistically significant when entered in the model (F = 1.66; B = -0.088; *p* = 0.188). Vulnerability group was significant in relation to quality of one’s life and health. The young group with psychosis rated their quality of life and health better than the recent immigrants with PTSD (F = 5.12; B = 6.48; *p* = 0.007) whereas no other group difference appeared. With vulnerability group in the model, activity level became a statistically significant positive factor as well (F = 4.04; B = 0.698; *p* = 0.047), together with activity satisfaction as a positive factor and recent work experience as a negative. Gender became non-significant in the model (F = 0.18; B = -0.647; *p* = 0.649), and in that step activity satisfaction (positively related) and recent work experience (negatively related) were the only activity factors that became statistically significant in relation to the quality of one’s life and health.

When age, vulnerability group, and gender were entered simultaneously in the model, vulnerability group was statistically significant, and activity satisfaction remained a positive factor and recent work experience a negative factor for the quality of life and health.

#### Quality of life: quality of the environment

The only activity factor that became statistically significant in relation to the quality of one’s environment in step 1 was activity satisfaction (F = 16.08; B = 0.137; *p* < 0.001).

None of the variables age, vulnerability group, or gender became statistically significant, and also not when entered together in step 3 of the model for quality of the environment.

#### Personal recovery

Higher scores on resources for having a worker role (F = 51.99; B = 0.992; p < 0.001) and on belief in having a worker role in the future (F = 10.68; B = 0.399; *p* = 0.007) were factors of significance for better ratings of personal recovery.

When age was entered in the model, it did not become significant. Type of vulnerability group and gender were also not significant in the model; nor were any of these factors when entered together.

#### Psychosocial functioning

Both activity level (F = 9.27; B = 1.264; *p* = 0.001) and resources for having a worker role (F = 8.67; B = 0.547; *p* = 0.004), but no other activity variable, showed statistically significant positive associations with psychosocial functioning in the initial model.

Older age (F = 6.51; B = 0.208; *p* = 0.018) was significantly associated with better psychosocial functioning, and when age was entered in the model the contribution of resources for having a worker role became non-significant. Type of vulnerability group was significantly associated with psychosocial functioning according to between-subjects effects when entered in the model, (F = 3.79; *p* = 0.025), but not according to parameter estimates (B = 5.157; *p* = 0.092). The tendency indicated was that the group with a history of SUD was rated as better functioning compared to the other two groups. Both activity level and resources for having a worker role were again statistically significant in the model (see Table 4). Gender was non-significant when entered in the model (F = 0.537; B = -1.260; *p* = 0.465).

When entered together, none of age, group or gender became significantly associated with psychosocial functioning. Activity level was the only activity factor with a statistically significant relation to psychosocial functioning, and resources for having a worker role again became non-significant.

#### Symptom level

Belief in a future worker role (F = 4.41; B = 0.326; *p* = 0.011) was the only activity variable with a statistically significant relationship with symptom level in step 1 of the model. Higher ratings were linked with less severe symptoms as assessed by the research assistant.

Age was non-significant in relation to symptom level (F = 1.34; B = 0.080; *p* = 0.250). Type of vulnerability became statistically significant according to statistics for between-subjects effects (F = 4.31; *p* = 0.016), but the beta values based on parameters estimates were non-significant (B = -0.417; *p* = 0.851 and B = 4.689; *p* = 0.065, respectively). The tendency was that the group with SUD was rated higher, thus as having less severe symptoms, compared to the other two groups. Gender entered separately with the activity variables did not become significant (F = 1.58; B = -1.778; *p* = 0.211), but inferred that the contribution from belief in a worker role to symptom level disappeared, and resources for having a worker role became statistically significant (F = 4.31; B = 0.324; *p* = 0.040). When age, group and gender were entered together, there was an interaction effect between vulnerability group and gender that became statistically significant in relation to symptom level (F = 4.20; *p* = 0.017), and no activity variable became statistically significant. Beta-values from parameter estimates were difficult to interpret for this interaction effect and the step 3 modelling was therefore split on the three vulnerability groups. These separate analyses indicated that in the group of young people with psychosis, female gender was associated with less severe symptoms (F = 8.23; B = -6.457; *p* = 0.007). Gender was non-significant in relation to symptom level in the two other groups.

## Discussion

This study aimed at identifying work and other activity factors of importance to well-being and functioning in three groups with mental illness, and exploring the importance of age, type of vulnerability and gender in relation to this. The bivariate correlations showed that each activity factor was associated with two or more of the factors addressing well-being and functioning. The GLM analyses, when all activity factors were taken into account simultaneously and the control factors were considered, gave a complementary and nuanced picture of relationships. The results discussed below are organized according to different dimensions of work and other activity: 1) future aspirations, 2) current situation, and 3) past experiences. While interspersing some of the contributions of the control factors (age, vulnerability group and gender), there is also a separate section addressing their roles.

### Future aspirations: beliefs in a worker role and in work resources

Belief in a future worker role was important for the life and health aspect of quality of life and, to a lesser degree, the environmental aspect. Both worker role aspects were strongly associated with personal recovery as well, which was obvious from both the bivariate analyses and the GLM models. These concordant findings suggest that belief in a future work role and resources for work are important for both quality of life and personal recovery, which is a finding that adds to previous knowledge. Previous research has shown that, compared to beliefs in a future worker role, having work resources was more closely related to well-being factors [34], but the current study could not corroborate that.

Resources for having a worker role was related with psychosocial functioning as well, but not when controlling for age, indicating that age was the stronger determinant of the two for level of psychosocial functioning. The finding that older age was linked with better functioning was not a product of vulnerability group, since the youngest group (the young people with psychosis) did not differ from the oldest (those with a history of SUD) on psychosocial functioning. The better-rated group was that consisting of immigrants with PTSD.

Belief in a future worker role and having work resources were also positively associated with level of psychiatric symptoms. This is in agreement with the criteria for assessment, where work is an important indicator [44], but may also be due to appropriate support in the PTSD center. Vulnerability group had an effect on the relation between ratings of the worker role and symptom level – resources for a worker role became significant instead – and when all control factors were entered together none of the activity variables became significant. This indicates that the function of the worker role factors for symptom level was fluctuating and ambiguous, possibly reflecting the varying impacts, both positive and negative, work may have on people’s state of mental illness [7, 8, 11].

### Current situation: actual work and other activities

Although belief in a future worker role was important for the life and health aspect of quality of life, according to the GLM model, recent work experience showed a negative relationship with the quality of life and health aspect, irrespective of control factors. This may seem like a surprising finding, but must be seen against the complexity inherent in salaried work, which is linked with both beneficial and detrimental attributes [49]. Work may entail positive experiences such as earning money, having work mates and contributing to society [6, 7], but also stigmatizing attitudes [11] and demands that can trigger mental ill-health [5]. It is plausible that those who had had a recent work experience had encountered negative experience and demands that were not in line with their capacities and needs, which would be in line with work by Honey [49], showing that whether employment constitutes a benefit or a drawback depends on both individual and contextual factors, and is dynamic over time.

Activity level became a positive factor for one’s quality of life and health, also when taking vulnerability group into account – the young group with psychosis had a better quality of life situation than those who were recent immigrants with PTSD. Swedish guidelines state that people with schizophrenia or similar conditions should receive support for engaging in meaningful everyday activity, including work and studies [50]. It is possible that the young people with psychosis, compared to the other two groups, received support that was more directed towards meaningful activity, which has been found to be associated with better quality of life among people with mental illness [51, 52]. Activity level was also associated with level of psychosocial functioning. This association is logical since the criteria for assessing level of functioning include activity factors such as work and engaging in everyday activities [44]. But despite some overlap, both reflect constructs in their own right; activity level addresses 12 additional activity areas and the assessment of psychosocial functioning includes several aspects other than work and activity, such as need of care from others, ability to perform rational acts, social withdrawal, and aggressive behavior [53].

The GLM model showed no association between actual work/studies or activity level and recovery, although the bivariate analyses showed a positive correlation between activity level and personal recovery. Our findings only lend moderate support towards work per se as being instrumental in promoting personal recovery in the three subgroups with mental illness under study and do not confirm the assumptions behind Supported Employment (SE) [54]. However, it should be noted that few SE studies have used recovery as an outcome and findings, although promising, are inconclusive [55]. A study interviewing people with mental illness about how they viewed work in relation to personal recovery concluded that whether work is beneficial or not for recovery depends on the personal meanings it brings to the person with mental illness [9].

### Past experiences: satisfaction with work and other activity

Activity satisfaction was particularly closely associated with quality of life, the health and life aspect as well as the environmental aspect. Bivariate associations showed that satisfaction with work experience was also moderately, positively associated with both aspects of quality of life, but became non-significant in the GLM model where it did not explain anything further in addition to activity satisfaction. This finding does not make satisfaction with work experience unimportant to quality of life and other aspects of well-being, however, as shown in previous research [3, 4]. In summary, the findings regarding satisfaction with past experiences are in agreement with research showing that subjective experiences from activity, such as the meaning [56], value [51] and engagement they generate, are closely associated with well-being.

### The roles of age, vulnerability group and gender

Vulnerability group was distinguished as the most important control factor, affecting several associations between activity factors and variables pertaining to well-being and functioning. This group variable added to the variation explained by activity factors, in relation to the life and health aspect of quality of life, psychosocial functioning, and symptom severity. None of the subgroups was, however, at risk of a worse situation for all of these. This is illustrated in Table 5, which includes only the well-being and function variables where vulnerability group had an effect according to the GLM models.

**Table 5:** The importance of vulnerability group according to the GLM analyses

	Quality of life and health	Psychosocial functioning	Symptom level
Young people with psychosis	**+**	**–**	**–**
People with a history of SUD	**0**	**–**	**+**
Recent immigrants with PTSD	**–**	**+**	**–**

*Note:* A positive position in relation to one or two other groups is indicated by plus (+), a negative by minus (−), and no statistically significant difference in relation to another group by zero (0)

The group of young people with psychosis had a better situation regarding quality of life and health in relation to one or both of the other groups, but worse in terms of psychosocial functioning and symptom level. The group with a history of SUD had an advantageous situation compared to both of the other groups regarding symptom level, but had a more detrimental situation in relation to the immigrants with PTSD regarding psychosocial functioning. Being an immigrant with PTSD was detrimental in relation to the young group with psychosis regarding the quality of one’s life and health, although they had a better situation compared to both of the other groups regarding level of psychosocial functioning.

Age was only relevant in relation to psychosocial functioning, suggesting that older age and experience was a positive factor. Sex was used as a variable to investigate possible gender effects in the associations between activity factors and variables addressing well-being and functioning. Gender formed an interaction effect with vulnerability group in the GLM model for symptom level. The fact that female gender was associated with less severe symptoms in the group of young people with psychosis aligns with research showing that men with schizophrenia and other psychoses tend to have more severe psychiatric symptoms [30]. Whether this is due to gendering of mental illness or an aspect of psychotic disorders per se may be debated. The fact that the onset is often earlier and more severe among men might explain the effect of gender in this study. Interestingly, however, a study involving > 400 psychiatrists who were asked to make a diagnosis based on a written case report, where 50% were informed that it was a man and 50% that is was a woman, found that the psychiatrists gave schizophrenia diagnosis more often to men than to women [57]. Another study concluded that gender may play a role in why men get more severe diagnoses, have a greater risk of being hospitalized and have longer stays in hospital [58]. Gender was not of importance in the groups of people with psychosis and recent immigrants with PTSD in the current study but is something that needs to be further studied in relation to mental illness.

#### Implications for practice

Satisfaction factors were more important to well-being and functioning in this study than were actual activities. Activity satisfaction stood out as a particularly important factor regarding quality of life. This means that support that aims to improve quality of life among people with mental illness should always consider the satisfaction these people perceive in relation to everyday activities. It is generally acknowledged that any activity can be therapeutic, as long as it is perceived as meaningful by the person [59]. On a similar note, research indicates positive relationships between community participation and recovery and quality of life, particularly if the participation is seen as important and in line with one’s values [60]. Inquiring about meaningful activities and providing opportunities for executing them and participating in the community would thus be a fruitful way of support towards better quality of life in groups similar to those included in the present study. Involving peer supporters [61] and using activity-based interventions [62–64] would be suitable measures in this respect.

Actual activity in terms of activity level was distinguished as closely linked with level of functioning, which should be considered when devising support in this respect. Belief in a future worker role and resources for having a worker role were above all strongly associated with personal recovery. This suggests that prospects regarding work should always be part of support to all groups with a mental illness. Focusing further on salaried work, this study clearly indicates that (better) support towards employment is imperative when attempting to support people with mental illness. Research on opportunities for work experiences among people with mental illness indicates, however, that the sociopolitical goal of fostering access to employment and work and to earning a living for this group is far from attained [65]. Nevertheless, most people with mental illness want salaried work and to make their own living [10], and research has concluded that people with various types of disabilities are an under-used resource in relation to the employment market [66, 67]. It is vital, however, as indicated in the present study, that the work experiences offered are perceived as meaningful and in line with the service user’s capacities and needs. Moreover, expectancies work both ways, and the expectations from workers and employers need to align.

#### Methodological considerations

The sampling for this study was not only based on diagnosis, but also on additional type of vulnerability. This would contribute to the internal validity of this study, since diagnosis per se has not been shown to play a role in research on relationships between everyday activity and well-being among people with mental illness, or psychosocial support to this group, [68, 69]. It is undoubtedly more common with research on diagnostic groups, and our focus on vulnerability type would supplement studies where sampling is based on diagnosis only. Regarding the group with SUD, these participants were approached since they were considered by staff to have a mental health illness combined with SUD. However, many of the participants did not report having a mental illness. This may be because the diagnosing of mental illness was made some years back in time and that SUD was currently the overshadowing problem in their own opinion.

As mentioned in the methods section, this study was under-powered, mainly due to a reorganization of the PTSD center and the fact that many possibly eligible young people had not yet had their diagnosis established. This means that there is a risk of Type-II errors and that true relationships were not detected. The strength of the identified statistically significant relationships may also have been underestimated. Intercollinearity may be another issue when several covariates are entered simultaneously in GLM analyses. A check of intercorrelations between variables entered as covariates revealed mainly weak or moderate associations, but there were a few relationships in the lower realm of strong (r_s_ < 0.6) according to the cut-offs proposed by Cohen [47]. For example, the association between resources for having a worker role and belief in a future worker role was in that span, but the fact that both became statistically significant when entered together indicates that intercollinearity was not a problem. Related to the issue of intercorrelations, the constructs behind activity satisfaction (SDO) and the quality of life variables (MANSA) overlap to some extent by the mere similarity among items. This concerns satisfaction with work and satisfaction with leisure, which are addressed in 2 of the 12 MANSA items and appear also in the SDO. It should further be noted that this study could not reveal any cause-effect relationships. Just as the activity variables influenced well-being and functioning, there was most likely an influence in the other direction as well. With respect to external validity, the findings should be possible to generalize to similar groups, but not to vulnerable groups in general.

This study could not include all aspects of relevance for well-being. For example, addressing social capital as well might have added value to the study since it has been found to contribute to well-being, alongside meaningful activities, among people with mental illness [70]. Race is another example, and might have been of relevance particularly for the recent immigrants with PTSD. Studies often raise a number of new questions, and further research is certainly needed, especially qualitative studies addressing the complexity of vulnerability in relation to work, other activities and well-being, as well as the type of support needed and wanted in different vulnerable groups with mental illness.

## Conclusion

Work and other activity variables explained significant proportions in quality of life and recovery, and to some extent in level of functioning. What stood out in particular was that activity satisfaction was associated with quality of life and that belief in a future worker role and having resources for that were of significance for personal recovery. A striking finding from this study is that recent work experience was detrimental to the life and health aspect of quality of life, while activity satisfaction was positively associated. This suggests that individuals may have expectations on work that are not met, and that a work experience in and of itself may not be conducive to well-being; it is satisfaction with work and other activities that matters.

Focusing on the selected control variables, age and gender were of minor importance. It was interesting though, that there was an effect of gender in the group of young people with psychosis, showing that women had less severe symptoms. This aligns with other research addressing psychosis and gender, and this topic should be further explored in future research. Vulnerability group mattered and showed to explain additional variation in both well-being and functioning. Which group was advantaged or disadvantaged in relation to the other groups varied, however, and no group seemed consistently more vulnerable than the other. Centering on identified strengths in the respective groups, the group of young people with psychosis perceived a higher level of quality of life; those with a history of SUD had less severe psychiatric symptoms; and the recent immigrants with PTSD had the highest level of psychosocial functioning. That such differences exist between subgroups of people with mental illness is vital to acknowledge in activity-based care and rehabilitation that aims to support people’s well-being and level of functioning. Qualitative studies could shed further light on how type of vulnerability entails possible additional difficulties and how that should be taken into consideration when devising mental health care and support.

## Supplementary Information

**Additional file 1.** Background Questionnaire.

## Data Availability

The data sets are not publicly available due to the restriction set by the Swedish Act concerning the Ethical Review of Research Involving Humans but are available from the corresponding author on reasonable request.

## Abbreviations

GLM: General Linear Modeling
PTSD: Post-traumatic Stress Syndrome
SUD: Substance Use Disorder
